# Metformin Induces PRODH/POX-Dependent Apoptosis in Breast Cancer Cells

**DOI:** 10.3389/fmolb.2022.869413

**Published:** 2022-06-06

**Authors:** Thi Yen Ly Huynh, Ilona Oscilowska, Lukasz Szoka, Ewelina Piktel, Weronika Baszanowska, Katarzyna Bielawska, Robert Bucki, Wojciech Miltyk, Jerzy Palka

**Affiliations:** ^1^ Department of Medicinal Chemistry, Faculty of Pharmacy, Medical University of Bialystok, Bialystok, Poland; ^2^ Department of Pharmaceutical and Biopharmaceutical Analysis, Faculty of Pharmacy, Medical University of Bialystok, Bialystok, Poland; ^3^ Department of Medical Microbiology and Nanobiomedical Engineering, Medical University of Bialystok, Bialystok, Poland

**Keywords:** metformin, proline dehydrogenase, proline oxidase, PRODH/POX, breast cancer cells, apoptosis

## Abstract

Although the antineoplastic activity of metformin (MET) is well established, the underlying mechanism of the activity is not understood. Since MET activates AMP kinase (AMPK) and proline dehydrogenase/proline oxidase (PRODH/POX) is stimulated by AMPK ligands (implicated in the regulation of cancer cell survival/apoptosis), the effect of MET on PRODH/POX-dependent apoptosis in wild-type MCF-7 cells (MCF-7^WT^) and POX knockdown MCF-7 cells (MCF-7^crPOX^ cells) was studied. PRODH/POX catalyzes proline degradation generating ROS-induced apoptosis or autophagy. Availability of proline for PRODH/POX functions is regulated by the activity of prolidase (enzyme releasing proline from imidodipeptides), collagen biosynthesis (process consuming proline), and metabolism of proline, ornithine, and glutamic acid. We have found that MET is cytotoxic for MCF-7 cells (IC50∼17 mM), and to the lower extent for MCF-7^crPOX^ cells (IC50∼28 mM). In MCF-7^WT^ cells, the effect was accompanied by the inhibition of DNA biosynthesis, collagen biosynthesis, stimulation of ROS formation, AMPKα phosphorylation, and expression of prolidase, p53, caspase 8, caspase 9, and cleaved PARP. In MET-treated MCF-7^crPOX^ cells, the processes were less affected than in MCF-7^WT^ cells and the expression of caspase 9 was decreased, while cleaved caspase 8 and cleaved PARP were not detected. The effects were accompanied by an increase in the prolidase activity and proline concentration. The mechanism for MET-induced apoptosis involves the up-regulation of prolidase activity and a decrease in collagen biosynthesis contributing to an increase in the concentration of substrate (proline) for PRODH/POX-dependent ROS formation and activation of caspases −9 and −8. The data suggest that PRODH/POX participates in the MET-induced intrinsic and extrinsic apoptosis in MCF-7 cells.

## Introduction

Metformin (MET) is widely used to treat type II diabetes. It evokes multidirectional activity by a decrease in the intestinal glucose absorption, the inhibition of gluconeogenesis, an increase in glycolysis, tissue sensitivity to insulin, and hypoglycemia (Rena et al., 2017). However, the most intriguing property of MET is its anti-cancer activity. Pharmaco-epidemiological studies initiated in 2005 provided evidence for a reduced risk of different cancers in diabetic patients receiving MET (Evans et al., 2005). Recently, several clinical trials were conducted on the antineoplastic potency of MET (Chae et al., 2016; De Flora et al., 2016). However, the mechanism of anti-cancer potential of MET is still unknown. It has been considered that the underlying mechanism of anti-cancer potential of MET could be due to the activation of AMP-activated protein kinase (AMPK) (Salani et al., 2012; Han et al., 2013; Wang et al., 2015; Guo et al., 2016) and inhibition of mitochondrial respiration (Rena et al., 2017).

Activation of AMPK through its phosphorylation takes place in conditions of energy shortage (e.g., starvation) when the ratio of AMP/ATP increases. This kinase, in order to restore the proper level of ATP, stimulates oxidative phosphorylation and inhibits the expenditure of energy, e.g., for cell proliferation (Hardie, 2003; Gwinn et al., 2008). The overall function of AMPK is the inhibition of anabolic processes and stimulation catabolism. Under conditions of energy shortage caused by defects in carbohydrate metabolism, an alternative energy source is proline. This amino acid could come from protein degradation, mainly from collagen, the protein containing the highest amount of proline among all proteins. This unique amino acid is oxidized by the mitochondrial enzyme proline dehydrogenase/proline oxidase (PRODH/POX) (Liu and Phang, 2012; Phang et al., 2012). Of interest is that the enzyme expression is up-regulated by AMPK (Pandhare et al., 2009).

PRODH/POX catalyzes the conversion of proline to ∆1-pyrroline-5-carboxylate (P5C). This process yields electrons that could be transported to the electron transport chain producing ATP-promoting cell survival (Donald et al., 2001; Phang et al., 2008a; Liu and Phang, 2012) or they could be accepted by oxygen, producing reactive oxygen species (ROS) to promote apoptosis/autophagy (Raha and Robinson, 2001; Martindale and Holbrook, 2002; Liu et al., 2006; Hu et al., 2007). Therefore, PRODH/POX may play a dual role, but the mechanism that switches PRODH/POX from cell growth supporting the growth inhibiting factor is unknown. The critical factor in this switching process is the availability of proline as a substrate for PRODH/POX-dependent functions. The main sources of intracellular proline are collagen degradation products (CDPs) (Jackson et al., 1975). Extracellular collagen degradation initiated by metalloproteinases is completed intracellularly. CDPs are hydrolyzed in lysosomes yielding free amino acids and imidodipeptides (e.g., glycyl-proline) that are further cleaved in cytoplasm by prolidase (E.C.3.4.13.9). Prolidase, known also as peptidase D or imidopeptidase, cleaves imidodipeptides with proline or hydroxyproline at the C-terminal position (Myara et al., 1984; Mock et al., 1990; Palka and Phang, 1997). The enzyme significantly contributes to the regulation of free proline concentration in cytoplasm (Myara et al., 1984; Palka and Phang, 1997; Surazynski et al., 2008).

Another factor regulating the intracellular concentration of proline is collagen biosynthesis (Priest and Davies, 1969). Synthesis of this protein requires a large amount of proline that constitutes about 25% of all amino acids building collagen molecules. In this context, collagen biosynthesis functions as a sink for proline, decreasing its concentration in cytoplasm and availability for PRODH/POX.

It has been suggested that the PRODH/POX-dependent generation of ROS may contribute to the initiation of intrinsic and extrinsic apoptotic pathways (Raha and Robinson, 2001; Martindale and Holbrook, 2002; Liu et al., 2006; Hu et al., 2007; Phang et al., 2008a). PRODH/POX overexpression causes the leakage of cytochrome c from mitochondrion to cytoplasm and the activation of caspase-9 and caspase-3 inducing intrinsic apoptosis (Liu et al., 2006). Interestingly, in the same conditions (overexpression of PRODH/POX), the extrinsic apoptotic pathway involving TNF-related apoptosis inducing ligand (TRAIL), death receptor 5 (DR5), and an increase in the expression of cleaved caspase-8 was observed (Liu et al., 2006).

However, PRODH/POX-mediated apoptosis could occur through different mechanisms in different cancer cells. It may involve the deregulation of cell signaling and cell cycle regulatory pathways. One of the most potent PRODH/POX activators is protein p53. PRODH/POX could be therefore transcriptionally regulated by p53 since the promoter of PRODH/POX contains a specific p53-response element (Polyak et al., 1997; Phang et al., 2008b; Maxwell and Kochevar, 2008).

The aim of this study is the identification of the mechanism of anticancer activity of MET in breast cancer cells. We evaluated the effect of MET on the expression of AMPK, PRODH/POX, cytoplasmic concentration of proline, collagen biosynthesis, and some apoptosis-inducing proteins in MCF-7 and PRODH/POX knockdown MCF-7 cells (MCF-7^crPOX^ cells).

## Materials and Methods

### PRODH/POX Knockout CRISPR-Cas9 DNA Plasmid Purification

The sgRNAs for PRODH/POX (CRISPR All-In-One Non-Viral Vector with spCas9) were products of ABM Company. The vector containing expression construct was transfected into *Escherichia coli* DH5α cultured in Luria-Bertani (LB) medium with 100 μg/ml ampicillin for 24 h at room temperature. The extraction of the targeted plasmid was performed using a plasmid DNA purification kit (Nucleobond Xtra Midi/Maxi, MACHERY-NAREL GmbH). The purified samples were precipitated with isopropanol, washed with 70% ethanol, and submitted to DNA cleaning-up step by the GeneMATRIX Basic DNA Purification Kit (EURX, E3545-01 protocol 1). DNA concentration was evaluated by NanoDrop™ 2000/2000c Spectrophotometer.

### Transfection Into MCF-7 Breast Cancer Cell Line

MCF-7 cells were cultured in DMEM 1X (Gibco), containing 10% fetal bovine serum (FBS) qualified (Gibco), 4.5 g/L glucose, L-glutamine, and pyruvate supplemented with 1% penicillin/streptomycin (Invivogen) at 37°C in 5% CO_2_. The cells were cultured in 6-well plates to achieve about 70–90% of confluency. The amount of plasmid tested in the experiment was 1–2 µg/well. For transfection, lipofectamine 2000 (Invitrogen) was used.

Prior to transfection, the plasmid was diluted with 50 µL of medium A, DMEM 1X (Gibco) without FBS. The transfection solution (805.4 µL of medium A and 194.6 µL of lipofectamine) was gently mixed and incubated for 5 min at room temperature. The cells tested were washed with PBS 1X (sterile, Gibco), 1 ml of medium A was added and incubated for 20 min, and then the mixture of plasmid with transfection reagent was added to cells and incubated overnight at 37°C in 5% CO_2_. The next day, the transfected cells were selected in the complete growth medium with 1 μg/ml of puromycin (Sigma-Aldrich) in the same culture conditions for 10 days. The PRODH/POX expression was analyzed by Western blot. Based on the expression level, the PRODH/POX knockout MCF-7 cell line was selected for further stable clone generation. The screening steps were done with a random selection of cell clones. The efficiency of PRODH/POX silencing was measured by Western blot using an anti-PRODH/POX antibody (Santa Cruz). The PRODH/POX knockout MCF-7 cells defined as MCF-7^crPOX^ cells were banked for further experiments.

### Cell Culture

The MCF-7 breast cancer cells (HTB-22) were purchased from ATCC Company (Manassas, United States). Wild-type MCF-7 cells (MCF-7^WT^) and MCF-^7crPOX^ cells were cultured in DMEM with 10% fetal bovine serum, 50 IU/ml penicillin, and 50 μg/ml streptomycin at 37°C in 5% CO_2_. The medium was changed every 3 days until the cells reached 80% of confluency. Then, the cells were treated for 48 h with MET at a concentration of 0–80 mM in glutamine-free medium.

### Cell Viability

The cell viability was measured by the MTT assay, as described previously (Zareba et al., 2018). The control and treated cells were washed twice with PBS, and a working solution of MTT at a final concentration of 0.5 mg/ml was added and the cells were incubated for 4 h. The formed formazan precipitate was dissolved in DMSO, and absorbance was measured at 540 nm (Varioscan Lux, Thermo Fisher Scientific, Waltham, MA, United States). The cell survival was calculated as a percentage of control.

### DNA Biosynthesis

The assay of DNA biosynthesis was based on the evaluation of (methyl-3^H^]-thymidine incorporation in the DNA of proliferating cells, as described previously (Zareba et al., 2018). The cells at 80% of confluency were treated for 44 h with MET in the medium without glutamine and next for 4 h with 0.5 μCi/ml of (methyl-3^H^]-thymidine. Incorporation of radioactive thymidine into DNA was evaluated by a Liquid Scintillation Analyzer Tri-Carb 2810 TR with Quanto SMART™ software.

### Collagen Biosynthesis

The assay for collagen biosynthesis was based on the evaluation of 5 (^3^H]-proline (5 μCi/ml) incorporation into proteins susceptible to *Clostridium histolyticum* collagenase, as described previously (Huynh et al., 2020). The cells were cultured in conditions described above for 48 h. Incorporation of radioactive proline was assessed in compliance with the method of Peterkofsky et al. (Peterkofsky and Diegelmann, 1971). Incorporation of a radioactive tracer was evaluated by a Liquid Scintillation Analyzer Tri-Carb 2810 TR with Quanto SMART™ software. Data are shown as cumulative values for cell and medium fractions.

### Determination of Prolidase Activity

The enzyme activity was measured by the colorimetry method of Myara et al. (Myara et al., 1982) based on the measurement of proline release from synthetic substrate, glycyl-proline. The activity of the enzyme was presented as an amount of proline (nmoles) released from the substrate for 1 min per milligram of the supernatant protein in cell homogenate. The protein was measured by the method of Lowry et al. (Lowry et al., 1951)

### Western Blot

Proteins were analyzed by Western blot, as described previously (Zareba et al., 2017; Zareba et al., 2018). The cell lysates containing 20 µg of protein were submitted to SDS-PAGE (10% polyacrylamide gel) for 1 h in room temperature. After transferring proteins to membranes, they were blocked and incubated with selected antibodies (Leśniewska et al., 2009): rabbit anti-caspase-9, anti-caspase-8, anti-cleaved-caspase-8, anti-p53, anti-pS46-p53, anti-PARP, anti-cleaved-PARP, anti-AMPKα, anti-pAMPKα, anti-PEPD, anti-collagen type IV, anti-collagen type VI, anti-ERK1/2, anti-phospho-ERK1/2, and goat-anti-PRODH, diluted 1:1000 in a blocking buffer. Then, the membranes were washed in TBS with 0.05% Tween (TBST) 3 × 15 min and incubated with the respective HRP-linked secondary antibody at a concentration of 1:7500 for 60 min at RT with gentle agitation. After washing in TBS-T (5 × 5 min), the membranes were incubated with an Amersham ECL Western Blotting Detection Reagent. Pictures were taken using a BioSpectrum Imaging System UVP. Densitometry analysis was performed; the mean values of band intensity are presented in Figures 3, 4.

### LC–MS-Based Quantitative Analysis

Quantitative analysis of proline was performed using a 1260 Infinity HPLC coupled to a 6530 Q-TOF mass spectrometer equipped with a dual ESI source (Agilent Technologies, Santa Clara, CA, United States) in the positive ionization mode according to the method of Klupczynska et al. (Klupczynska et al., 2020). Chromatographic separation was conducted on a Luna HILIC column (2.0 × 100 mm, 3 μm particle size, Phenomenex, Torrance, CA, United States). The total protein concentration was used for normalization. The data were presented as a percent of control values.

### ROS Generation Assessment

Accumulation of ROS was assessed by using DCFH-DA (fluorescent probe), as described previously (Huynh et al., 2020). The cells were pre-treated with DCFH-DA (20 µM) for 30 min, washed by PBS, and incubated with 20 mM MET for 48 h in DMEM without glutamine. The intensity of fluorescence was measured at an excitation/emission wavelength of 488/535 nm using a TECAN Infinite® M200 PRO (Männedorf, Switzerland). The results were presented as a percent of control values.

### Apoptosis Induction

The translocation of phosphatidylserine (PS) to the cell surface due to MET treatment was measured using the Muse® Annexin V and Dead Cell Kit (Merck, Germany). The combination of FITC-labeled Annexin V with 7-AAD (7-aminoactinomycin D; indicator of cell membrane structural integrity) allowed us to distinguish the apoptotic cells. For the purpose of the clarity of presented data, live cells (Annexin V-negative and 7-AAD-negative) were not presented in the provided figures.

### Immunofluorescence Microscopy

Immunostaining was conducted according to the BDB Bioimaging protocol, as described previously (Zareba et al., 2017). Cells exposed to MET (20 mM) were fixed with paraformaldehyde, permeabilized with Triton, blocked with 3% FBS, and incubated with anti-cleaved-caspase-7 (primary antibodies) and FITC Fluor-conjugated secondary antibody and Hoechst. Samples were visualized with a confocal laser scanning microscope (BD Pathway 855 Bioimager) using AttoVision software.

### Statistical Analysis

All experiments were carried out in duplicates, and the experiments were repeated at least three times. Data are shown as a mean ± standard deviation (SD). For statistical calculations, a one-way analysis of variance (ANOVA) with Dunnett’s correction and t-test was used. Statistical analysis was performed using GraphPad Prism 5.01 (GraphPad Software, San Diego, CA, United States). Statistically significant differences were marked as *p < 0.01 and **p < 0.001.

## Results

**FIGURE 1:**
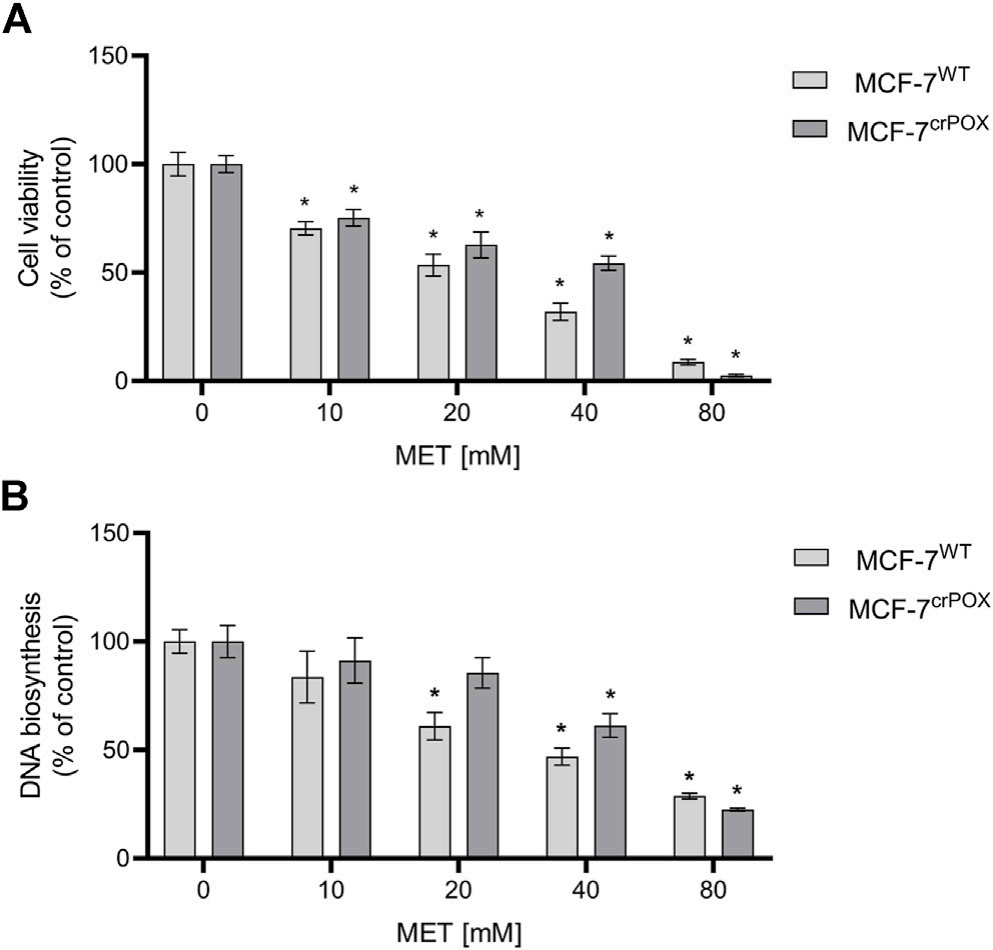
Cell viability **(A)** and DNA biosynthesis **(B)** in wild-type MCF-7 cells (MCF-7^WT^) and knocked-down PRODH/POX MCF-7 cells (MCF-7^crPOX^) upon treatment with indicated concentrations of metformin (MET) for 48 h in glutamine-free medium. The mean values with standard deviation (SD) from three experiments performed in duplicates are presented. Asterisks (*) indicate statistical differences between studied cells compared to controls at *p* < 0.01.

MET cytotoxicity in numerous breast cancer cell lines is a well-established phenomenon (Effect of Metformin on Breast Cancer Metabolism, 2014; Metformin clinical trial, 2014). To test whether PRODH/POX is involved in MET-induced cytotoxicity, studies were performed on wild-type MCF-7 cell line (MCF-7^WT^), expressing PRODH/POX and on PRODH/POX knocked out cells (MCF-7^crPOX^) generated by the CRISPR/Cas9 method. The effect of MET on the cell viability in both cell lines was measured by the MTT assay (Figure 1A). Cells were incubated with MET for 48 h. The IC_50_ value for MCF-7^WT^ cells was 16.61 mM and for MCF-7^crPOX^, it was 27.61 mM. The data were corroborated by the results on DNA biosynthesis showing a dose-dependent inhibitory effect of MET on the process (Figure 1B). The data suggest that MET-induced cytotoxicity in MCF-7 cells could be partially PRODH/POX-dependent.

**FIGURE 2:**
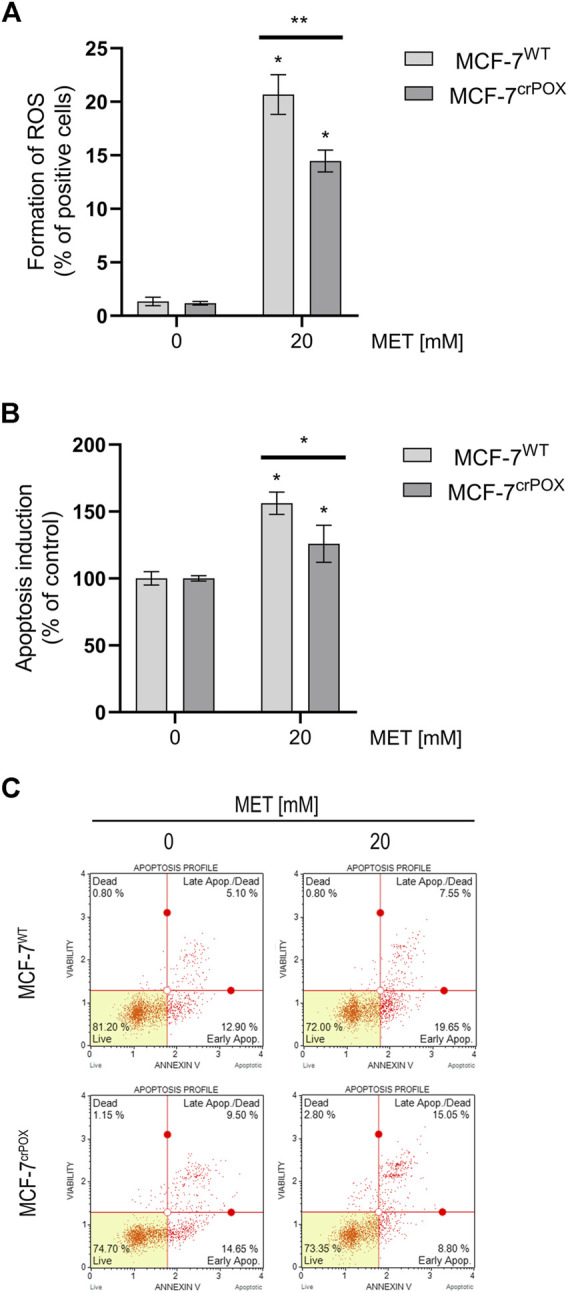
ROS formation **(A)**, apoptosis induction **(B)**, and representative plots of flow-cytometry-based analysis of Annexin V **(C)** in wild-type MCF-7 cells (MCF-7^WT^) and knocked-down PRODH/POX MCF-7 cells (MCF-7^crPOX^) treated with 20 mM metformin (MET) for 48 h in glutamine-free medium. The mean values with standard deviation (SD) from three experiments performed in duplicates are presented. Asterisks (*) indicate statistical differences between studied cells compared to controls at *p < 0.01 and **p < 0.001.

Since in some experimental conditions PRODH/POX is known to induce the production of ROS, the effect of MET on the process was studied using dichlorodihydrofluorescein diacetate (DCFH-DA), a fluorogenic dye that when oxidized by ROS produces fluorescent dichlorofluorescein (DCF). Cells were treated with 20 mM of MET, a concentration that decreased the viability of MCF-7^WT^ cells almost by a half. It has been found that the treatment of MCF-7^WT^ with MET for 48 h increased ROS generation by about 20-fold, compared to that of control cells. In MCF-7^crPOX^ cells, MET treatment in the same conditions increased ROS production by about 15 times, compared to control (Figure 2A). It suggests that MET-dependent ROS generation is partially dependent on PRODH/POX.

Generation of ROS may lead to apoptosis; therefore, the process was assessed by double-staining with annexin V and 7-AAD in both cell lines. Treatment of cells with 20 mM MET for 48 h increased the percentage of apoptotic cells in MCF-7^WT^ by about 50%, while in MCF-7^crPOX^, it increased only by about 25%, compared to non-treated cells (Figure 2B). It has been confirmed by results from the flow-cytometry-based analysis of Annexin V in MET-treated cells (Figure 2C). MCF-7^WT^ and MCF-7^crPOX^ cells were analyzed by flow cytometry using Annexin V-FITC/7-AAD double staining. A combination of Annexin V and 7-AAD allowed the cells to be sorted into four groups (presented in Figure 2C): early apoptotic cells [Annexin V (+)/7-AAD (–)], late apoptotic/dead cells [Annexin V (+)/7-AAD (+)], dead cells [Annexin V (–)/7-AAD (+)], and live cells [Annexin V (–)/7-AAD (–)]. The calculated percentages of apoptotic and dead cells are presented in Figure 2B. In the cells treated with 20 mM of MET, a significant number of cells were in the late-stage apoptosis in both cell lines. Moreover, it was noted that some of these cells were still undergoing early apoptosis since they were classified as Annexin V-positive/7-AAD-negative. Collectively, these results clearly indicate that MET-based treatment induces apoptosis in breast cancer cells. It suggests a significant role of PRODH/POX in MET-dependent apoptosis in MCF-7^WT^ cells.

**FIGURE 3:**
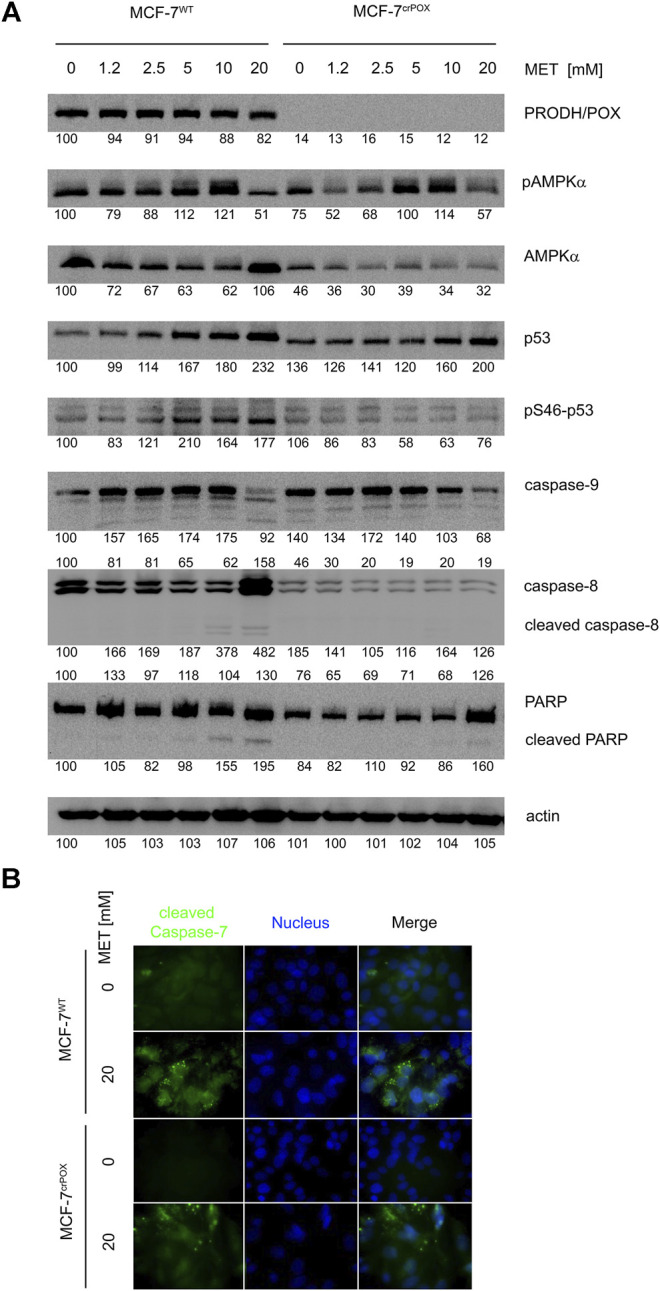
Western blot of some proteins involved in proline metabolism and apoptosis **(A)** and immunofluorescence bioimaging of cleaved caspase-7 **(B)** in wild-type MCF-7 cells (MCF-7^WT^) and knocked-down PRODH/POX MCF-7 cells (MCF-7^crPOX^) treated with metformin (MET, 0–20 mM) for 48 h in glutamine-free medium. Representative blot images are shown (the mean value of the densitometric analysis of protein bands is presented below each blot).

In order to evaluate the mechanism of MET-dependent apoptosis and the role of PRODH/POX in apoptosis, the expression of some proteins involved in this process was determined by Western blot (Figure 3A).

MET did not affect the PRODH/POX expression in MCF-7^WT^ cells, indicating that the ability of the cells to oxidize proline is also preserved during MET-induced apoptosis.

MET has been identified as a potent stimulator of AMP-dependent kinase (AMPK). The enzyme is a heterotrimer composed of alpha, beta, and gamma subunits. The alpha subunit is post-translationally activated by phosphorylation. In both cell lines treated with MET, an increase in phosphorylation of the *α*-subunit of AMPK was observed. One of the substrates of the AMP-dependent kinase is the p53 protein. In MCF-7^WT^ cells, we found a parallel increase in the phosphorylation of AMPK and the S46 serine residue located in the p53 transactivation domain. However, this change was not greater than the increase in the p53 (total) protein expression due to MET-treatment. In MCF-7^crPOX^ cells, the phenomenon was not observed.

The p53 protein is a key factor in activating apoptosis in response to DNA damage. MET-induced increase in the p53 expression in both cell lines was accompanied by a decrease in the expression of procaspase-9 and the absence of cleaved caspase-8. Expression of caspase 8 was induced by 20 mM MET only in MCF-7^WT^ cells. Changes in the expression of initiator caspases are accompanied by the cleavage of PARP—one of the caspase 3 substrates. In both cell lines, MET induced the expression of PARP; however, the cleaved PARP was found only in MCF-7^WT^ cells treated with 10 and 20 mM MET. The observed changes in the protein expression confirm the activation of apoptosis in MCF-7^WT^ cells treated with MET. The results were confirmed by immunofluorescence bioimaging of caspase-7 in studied cells (Figure 3B).

**FIGURE 4:**
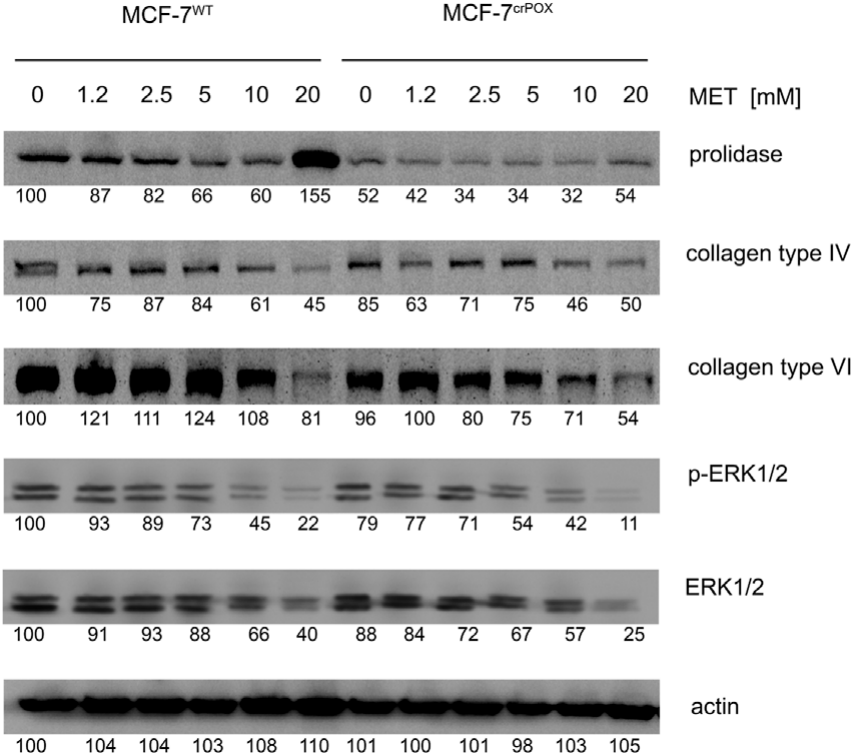
Western blot for prolidase, collagen types IV and VI, and ERK1/2 in wild-type MCF-7 cells (MCF-7^WT^) and knocked-down PRODH/POX MCF-7 cells (MCF-7^crPOX^) treated with metformin (MET, 0–20 mM) for 48 h in glutamine-free medium. Representative blot images are shown (the mean value of the densitometric analysis of protein bands is presented below each blot).

As presented in Figure 4, MET contributed to a decrease in the expression of collagen (e.g., types IV and VI) in both cell lines (MCF-7^WT^ and MCF-7^crPOX^). The biosynthesis of collagen requires a large supply of proline; therefore, when the process is reduced, it supports free proline for PRODH/POX-dependent functions. At 20 mM, MET induced the expression of prolidase, an enzyme catalyzing the recovery of proline from imidodipeptides (degradation products of collagen) in MCF-7^WT^ cells. The process was less pronounced in MCF-7^crPOX^ cells. An increase in the prolidase expression should result in the increase in the intracellular proline concentration, thus providing a substrate for PRODH/POX. These MET-dependent processes were accompanied by a dose-dependent decrease in the expression of ERK1/2 in both cell lines.

**FIGURE 5:**
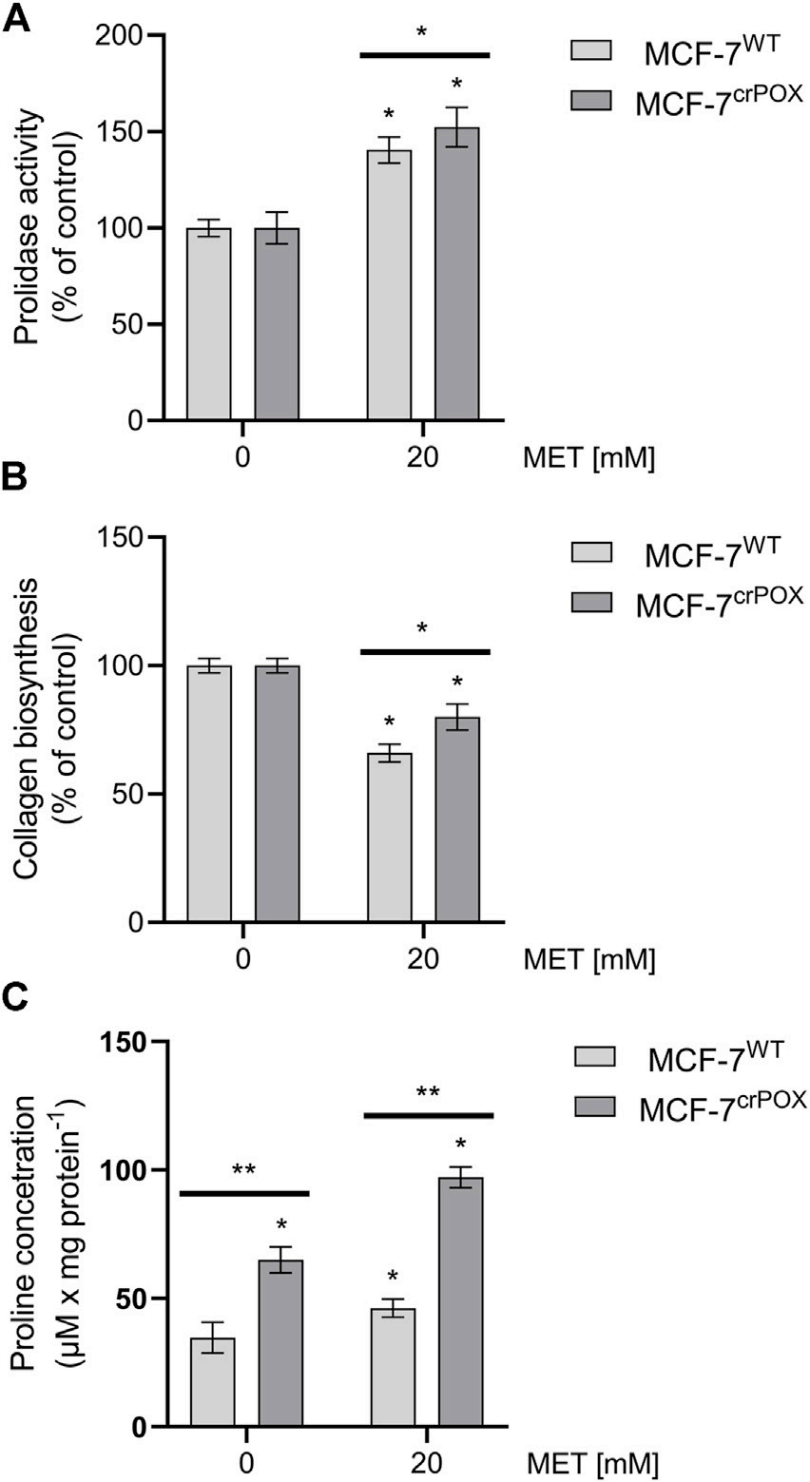
Prolidase activity **(A)**, collagen biosynthesis **(B)**, and proline concentration **(C)** in wild-type MCF-7 cells (MCF-7^WT^) and knocked-down PRODH/POX MCF-7 cells (MCF-7^crPOX^) upon treatment with 20 mM metformin (MET) for 48 h in glutamine-free medium. The mean values with standard deviation (SD) from three experiments performed in duplicates are presented. Asterisks (*) indicate statistical differences between studied cells compared to controls at *p < 0.01 and **p < 0.001.

MET at 20 mM drastically increased not only prolidase expression (Figure 4) but also prolidase activity in MCF-7^WT^ cells and to a lesser extent in MCF-7^crPOX^ cells (Figure 5A). Similarly, MET down-regulated not only collagen types IV and VI expressions in both cell lines (Figure 3) but also the total collagen biosynthesis (Figure 5B). It suggests that despite PRODH/POX knockout, the MET ability to inhibit collagen biosynthesis was preserved. Thus, in MCF-7^crPOX^ cells, a decrease in the utilization of proline for protein biosynthesis and inability of proline to be oxidized (due to the lack of PRODH/POX) contributed to an increase in the concentration of proline in the cells (Figure 5C). The data suggest that MET-dependent increase in the prolidase activity and a decrease in collagen biosynthesis contributed to an increase in the intracellular concentration of proline, a substrate for PRODH/POX.

## Discussion

In this report, we provide evidence for the important role of PRODH/POX in the mechanism of MET-induced apoptosis in MCF-7 cells. We hypothesized that the sequence of events could involve i/MET-induced AMPK, ii/AMPK-induced PRODH/POX, and iii/PRODH/POX-dependent ROS generation inducing apoptosis. The hypothesis was based on previous reports showing that the overexpression of PRODH/POX contributed to apoptosis by intrinsic and extrinsic pathways (Raha and Robinson, 2001; Martindale and Holbrook, 2002; Liu et al., 2006; Hu et al., 2007; Phang et al., 2008a). However, the results presented in the present report show that MET did not stimulate the PRODH/POX expression. Therefore, it has been considered that proline availability for PRODH/POX could determine ROS generation with further consequences on apoptotic pathways. In fact, MET facilitated proline availability for PRODH/POX-dependent functions by the inhibition of collagen biosynthesis. The collagen biosynthesis inhibiting activity of MET was shown both in MCF-7^WT^ and in MCF-7^crPOX^ cells with a weaker inhibiting effect on the latter. It was accompanied by a significant increase in thw intracellular proline concentration in MCF-7^WT^ and a drastic increase in the concentration of this amino acid in MCF-7^crPOX^ cells. The rational explanation for the phenomenon is that PRODH/POX is the only proline degrading enzyme; therefore, the knockdown contributed to a drastic increase in intracellular proline accumulation. However, the level of free proline could not be considered as a contributor to the activation of apoptosis. The increase in proline concentration in MET-treated MCF-7^WT^ cells was much lower than in MET-treated MCF-7^crPOX^ cells, while MET was more cytotoxic for MCF-7^WT^ cells (IC_50_∼17 mM versus IC_50_∼28 mM for MCF-7^crPOX^ cells) and strongly induced apoptosis only in MCF-7^WT^ cells. It seems that the mechanism of MET-induced apoptosis could be attributed to the inhibition of collagen biosynthesis and an increase in proline availability for PRODH/POX and ROS generation. In this report, we have provided evidence that the treatment of MCF-7^WT^ cells with MET increased ROS formation and the expression of caspase 8, caspase 9, and cleaved PARP. In MET-treated MCF-7^crPOX^ cells, the processes were less affected.

The data suggest that both PRODH/POX and proline availability for the enzyme play an important role in the mechanism of MET-dependent apoptosis in MCF-7 cells. Since PRODH/POX is considered as an inhibitory factor in tumor progression (Donald et al., 2001; Maxwell and Rivera, 2003; Liu et al., 2005), the metabolism of its substrate, proline, in neoplastic cells is of great importance.

Several studies showed that in various cancers, PRODH/POX is down-regulated (Kononczuk et al., 2015a) and the proline concentration is increased (Hirayama et al., 2009; Catchpole et al., 2011). Although several mechanisms for down-regulation of PRODH/POX were described (Kononczuk et al., 2015a), the elevation of proline concentration in cancer cells is attributed to an increased degradation of type I collagen in the extracellular matrix (Ii et al., 2006). It usually happens in conditions of energy shortage accompanying cancer cell metabolism due to the Warburg effect. In fact, glucose depletion (Pandhare et al., 2009) was found to induce matrix metalloproteinases MMP-2 and MMP-9, suggesting that the increase in cellular proline concentration in cancer cells is a result of collagen degradation. However, proline could be synthesized from glutamine, the amino acid essential for cell growth (D'Aniello et al., 2020). Taking this fact into consideration, the studies presented in this report were performed in glutamine-free medium limiting its availability for proline synthesis. It creates conditions for the utilization of proline from other sources, e.g., collagen degradation. Moreover, this culture condition (energy shortage) facilitated the activation of AMPK and potentially PRODH/POX (Liu et al., 2012a; Phang and Liu, 2012). In conditions of energy shortage, cancer cells may prefer proline as an alternative energetic substrate. In *in vivo* conditions, proline has an advantage over fatty acids, glutamine, and glucose that require delivery by the circulation. In the presented conditions, proline could be directly used from extracellular matrix protein degradation. In this context, proline may serve as an energy substrate as well as an energetic sensor. In fact, in conditions of energy shortage, the cells adapt to the metabolic changes by activating AMPK (Laderoute et al., 2006; Wang and Guan, 2009), which inhibits energy-consuming processes and stimulates the production of energy to restore energy homeostasis (Kato et al., 2002; Hardie, 2008). It cannot be excluded that in such conditions, MET not only inhibits collagen biosynthesis but also induces collagen degradation.

However, according to our result, it seems that PRODH/POX is not necessary for maintaining a high level of AMPK. A high expression of PRODH/POX is usually accompanied by a high level of AMPK, since this kinase is a potent stimulator of PRODH/POX (Pandhare et al., 2009). Recently, we provided evidence that PRODH/POX knockout induced reprogramming of energetic metabolism of MCF-7 cells, suggesting that such a modification of the cells can affect pAMPK-α (Huynh et al., 2021). It seems that the decreased level of pAMPK in MCF7^crPOX^ cells, compared to MCF7^WT^ cells (Figure 3), is a result of such a mechanism. Whether there is a direct link between AMPK and PRODH/POX requires to be established.

Another explanation for the lower expression of pAMPK-α in MCF7^crPOX^ cells, compared to that in MCF7^WT^, comes from studies suggesting an interplay between AMPK and p53 in the mechanism of MET-dependent apoptosis in MCF-7 cells, suggesting that in certain conditions, MET could induce apoptosis by the AMPKα-independent pathway, favoring p53 signaling (Yenmis et al., 2021). Such a specific condition was described by Yi et al. showing that a low concentration of MET stimulates p53-dependent senescence, while a high concentration causes apoptosis (Yi et al., 2013). In fact, PRODH/POX could be transcriptionally regulated by p53 (Polyak et al., 1997; Phang et al., 2008b; Maxwell and Kochevar, 2008), and MET-induced PRODH/POX-dependent apoptosis in MCF-7^WT^ cells was also accompanied by an increase in the expression of p53 and its phosphorylation. Phosphorylation of p53 determines its transcriptional activity (Liu et al., 2008; Maxwell and Kochevar, 2008). The similar increase in the p53 expression, but not its phosphorylation, was found in MET-treated MCF-7^crPOX^ cells not showing a pro-apoptotic phenotype. It could not be explained by the p-53-dependent transcriptional regulation of PRODH/POX in MCF-7^WT^ cells (Maxwell and Kochevar, 2008) since the PRODH/POX expression was not affected in these conditions. MET-induced p53 expression in both cell lines suggests that the process is PRODH/POX-independent. However, in contrast to MCF-7^WT^ cells, in MCF-7^crPOX^ cells, MET lost the ability to phosphorylate p53 (Figure 3). The MET-dependent increase in the expression of p53 in both cell lines was accompanied by an increase in the expression of caspases-8 and -9 in MCF-7^WT^ cells, while in MCF-7^crPOX^ cells, the expressions were decreased. Similarly, MET induced PARP expression in both cell lines; however, the cleaved PARP was detected only in MCF-7^WT^ cells treated with 10 or 20 mM MET. It suggests that PRODH/POX is involved in the mechanism of MET-induced apoptosis; however, the mechanism of the process needs further study.

We suggest that the MET-dependent increase in intracellular proline concentration provides a substrate for PRODH/POX-induced ROS generation, with further consequences on the activation of intrinsic and extrinsic apoptotic pathways. PRODH/POX cooperates with P5C reductases (mitochondrial PYCR1/2 and cytosolic PYCRL) participating in proline shuttling between mitochondria and cytoplasm. The conversion of P5C into proline is linked to glucose metabolism by the pentose phosphate pathway, while the conversion of proline into P5C is coupled to the TCA cycle (Phang et al., 2008b; Pandhare et al., 2009; Liu and Phang, 2012; Phang et al., 2012). It is vital for the maintenance of redox balance in a cell due to participation of NADPH/NADH in the conversion of P5C to proline. However, in cancer cells due to the Warburg effect, the deregulation of glycolysis and TCA cycle could enforce proline cycle. The above data suggest that ROS could be generated by the massive conversion of proline into P5C by PRODH/POX and P5C to proline by mitochondrial PYCR (Liu et al., 2012b), instead of cytosolic PYCRL converting P5C to proline and utilized for collagen biosynthesis (Pandhare et al., 2009). Probably, the inhibition of collagen biosynthesis by MET inhibits proline utilization in this process, therefore the amino acid again is degraded by PRODH/POX and up-regulates proline cycle. Such a cycle of proline/P5C within the mitochondrion could be responsible for ROS-induced apoptosis. Therefore, we postulate that the critical step in the mechanism of MET-dependent apoptosis is the inhibition of proline utilization for collagen biosynthesis. Although the presented mechanism for the switch between ATP and ROS production by PRODH/POX in mitochondria requires further study, it is partially supported by data showing that 2-methoxyestradiol, an inhibitor of collagen biosynthesis (Gelse et al., 2008), facilitates apoptosis and autophagy in certain cancer cells, e.g., adenocarcinoma cells (Theron et al., 2013). The similar tendency was found in the breast cancer tissue, characterized by a high prolidase activity and a low collagen content (Cechowska-Pasko et al., 2006). In our previous studies, we found that Eptifibatide (ligand of α5β3 integrin) induced the increase in prolidase activity, upregulated the PRODH/POX expression, and inhibited the biosynthesis of collagen in MCF-7 cells, resulting in the activation of intrinsic apoptosis, as shown by the increase in the expression of cleaved caspase-9 (Kononczuk et al., 2015b).

However, in several of our previous studies, we found that the role of PRODH/POX in the apoptosis/survival of cancer cells depends on the metabolic context of a specific cell type. In one of our recent articles (Huynh et al., 2021), we provided evidence that the stimulation of the PRODH/POX expression by MET induced apoptosis in both WT and PRODH/POX knockout MCF-7 cells, however, only when cultured in the absence of glutamine, while the presence of glutamine facilitated the pro-survival phenotype of the cells. Metabolomic analysis suggested that glycolysis is tightly linked to glutamine and proline metabolism in these cells, creating metabolic conditions for energy production and proline availability for PRODH/POX-dependent functions. MET treatment of both cell lines (WT and PRODH/POX knockout MCF-7 cells) cultured in glutamine-free medium contributed to glucose starvation, facilitating pro-apoptotic phenotype of these cells, as detected by the increase in the expression of active caspase-7 and PARP. Caspase-7 is known as an executioner protein of apoptosis activated by caspase-8 (extrinsic pathway) and caspase-9 (intrinsic pathway). The data suggested that in PRODH/POX-expressing cells and PRODH/POX knockout cells, apoptosis undergoes a different mechanism. In fact, in both cell lines, we have found that MET inhibits ERK1/2 expression in a dose-dependent manner. Although the MET-dependent inhibition of ERK1/2 signaling in breast cancer cells is well established (Malki and Youssef, 2011), the similar effect of MET on PRODH/POX knocked out cells is not known. Recently, it has been suggested that in certain conditions, PRODH/POX knockout can induce ROS-independent apoptosis through the activation of extrinsic apoptotic pathways (Kazberuk et al., 2022a). It seems that the mechanism for the process in PRODH/POX knockout cells involves a drastic increase in the proline concentration (bearing reducing potential). This phenomenon occurs when both pathways of proline utilization are blocked, namely, PRODH/POX and collagen biosynthesis. The functional significance of such a mechanism in the experimental cancer treatment is currently investigated.

It has been considered that proline availability for PRODH/POX could be an important factor in the creation of pro-apoptotic phenotype of cancer cells. In melanoma cells, characterized by the intense biosynthesis of collagen (proline consuming process) PRODH/POX knockout did not induce apoptosis. It seems that in the melanoma cells with a high capacity to utilize proline for collagen biosynthesis, the proline concentration is not high enough to induce extrinsic apoptosis. However, the stimulation of PRODH/POX by MET in melanoma cells induced ROS-dependent apoptosis, while PRODH/POX knockout abolished the effect (Oscilowska et al., 2022). A similar effect was found in MCF-7 cells treated with non-steroidal anti-inflammatory drugs (Kazberuk et al., 2022b). Other studies on MCF-7 and MDA-MB-231 cells highlighted the role of estrogens and estrogen receptors (ERα and ERβ) in PRODH/POX-dependent apoptosis (Lewoniewska et al., 2021). We have found that the activation of PRODH/POX (by troglitazone) induced apoptosis only in the absence of estradiol or ERβ in MDA-MB-231 cells.

Therefore, we suggest that the role of PRODH/POX in apoptosis/survival in cancer cells is metabolic context-dependent and the process occurs through various mechanisms in different types of cancer cells. Obviously, it limits the clinical relevance of MET use in breast cancer. To date, a decreased risk of breast cancer was observed only in patients with type 2 diabetes, receiving MET on a long-term basis (Bodmer et al., 2010). Similarly, in diabetic patients with breast cancer, MET treatment reduced the risk of metastases; however, the benefit ratio was very low (Jacob et al., 2016), suggesting that some patients respond to the treatment, while others do not. Since the mechanism of MET participation in these processes is unknown, several approaches have been undertaken to explore the issue. The results presented in this report provide insight into the complexity of molecular mechanisms creating PRODH/POX-dependent and PRODH/POX-independent apoptosis in cancer cells.

The application value of the results presented in this article has obvious limitations due to *in vitro* studies that should be confirmed by *in vivo* experiments. Although cell line models have several limitations (e.g., inability to observe systemic phenomena), they are a powerful tool that offers several advantages. Certainly, the cell models allow a strict control of the conditions of the experiment in order to establish the critical factor affecting the studied processes in a relatively short time. They are especially helpful in the case of limited availability of clinical samples or *in vivo* models. Therefore, results on cell models allow us to predict the consequences of pharmacotherapeutic manipulation in humans and provide rational for clinical studies on dose-dependent effects. Different treatment regimens and combinations of therapies have been tested using cell lines that have yielded interesting and potentially promising results that some of them could have an application value.

In this report, we suggest that the mechanism for MET-induced apoptosis involves an increase in the prolidase activity and a decrease in collagen biosynthesis providing substrate (proline) for PRODH/POX-dependent ROS formation and activation of caspases-9 and -8. The data suggest that PRODH/POX participates in the MET-induced intrinsic and extrinsic apoptosis in MCF-7 cells.

## Conclusion

MET-induced apoptosis involves the up-regulation of PRODH/POX-dependent ROS formation, the activation of caspases-9 and -8, an increase in prolidase expression and activity, a decrease in collagen biosynthesis, and an increase in the proline concentration providing a substrate for PRODH/POX. The data suggest that PRODH/POX participates in the MET-induced intrinsic and extrinsic apoptosis in MCF-7 cells.

## Data Availability

The original contributions presented in the study are included in the article; further inquiries can be directed to the corresponding author.
